# Gallic Acid Protects from Sepsis-Induced Acute Lung Injury

**DOI:** 10.3390/cimb46010001

**Published:** 2023-12-19

**Authors:** Süleyman Kardaş, Osman Sezer Çınaroğlu, Ejder Saylav Bora, Oytun Erbaş

**Affiliations:** 1 Department of Emergency Medicine, Kızıltepe State Hospital, Mardin 47400, Türkiye; 2 Department of Emergency Medicine, Faculty of Medicine, Izmir Katip Çelebi University, Izmir 35270, Türkiye; drsezer@hotmail.com (O.S.Ç.); saylavbora@hotmail.com (E.S.B.); 3 Department of Physiology, Faculty of Medicine Demiroğlu Science University, Istanbul 34000, Türkiye; oytunerbas2012@gmail.com

**Keywords:** gallic acid, sepsis, acute lung injury, inflammation

## Abstract

Sepsis, a leading global cause of morbidity and mortality, involves multiple organ dysfunction syndromes driven by free radical-mediated processes. Uncontrolled inflammation in early sepsis stages can lead to acute lung injury (ALI). Activated leukocytes generate reactive oxygen species, contributing to sepsis development. Gallic acid, a phenolic compound, is known for its antimicrobial properties. This study aims to observe gallic acid’s protective and restorative effect on the lungs in an experimental sepsis model. Male Wistar albino rats were subjected to a feces intraperitoneal injection procedure (FIP) to induce sepsis. Four groups were formed: normal control, FIP alone, FIP with saline, and FIP with gallic acid. Gallic acid was administered intraperitoneally at 20 mg/kg/day. Blood samples were collected for biochemical analysis, and computed tomography assessed lung tissue histopathologically and radiologically. Gallic acid significantly decreased malondialdehyde, IL-6, IL-1β, TNF-α, CRP levels, oxidative stress, and inflammation indicators. Lactic acid levels decreased, suggesting improved tissue oxygenation. Histopathological examinations revealed reduced lung damage in the gallic-acid-treated group. Computed tomography confirmed lower lung density, indicating less severe inflammation. Arterial blood gas analysis demonstrated improved oxygenation in gallic-acid-treated rats. Gallic acid exhibited anti-inflammatory and antioxidant effects, reducing markers of systemic inflammation and oxidative stress. The findings support its potential to protect against ALI during sepsis. Comparable studies underline gallic acid’s anti-inflammatory properties in different tissues. Early administration of gallic acid in sepsis models demonstrated protective effects against ALI, emphasizing its potential as an adjunct therapy to mitigate adverse outcomes. The study proposes gallic acid to reduce mortality rates and decrease the need for mechanical ventilation during sepsis-induced ALI.

## 1. Introduction

Globally, sepsis is a common cause of death and morbidity; it is distinguished by the manifestation of multiple organ dysfunction syndromes [1]. The underlying mechanism by which this condition develops is through the interaction of free radicals, which possess the capacity to disrupt cellular components. This interaction results in the formation of lipoperoxidation, which in turn causes a decrease in the fluidity of the cell membranes and ultimately advances the development of multiple organ failure [2,3,4,5]. Sepsis is more susceptible to developing in those with compromised immune systems and resistance to antimicrobial medicines [6]. A dysregulated cytokine response and an uncontrolled hyperinflammatory response are hallmarks of the early stages of sepsis [6]. Therefore, in the initial phases of sepsis, it is critical to regulate the inflammatory response to prevent organ damage that could ultimately result in organ failure and death.

Furthermore, existing documentation supports that activated leukocytes can produce reactive oxygen species [ROS] [7,8]. The overstimulation of the respiratory burst, a process associated with several medical conditions, including asthma, diabetes, Parkinson’s disease, and Alzheimer’s disease [9,10,11], can play a role in the onset of sepsis.

Acute lung injury (ALI) is a severe and debilitating consequence that can arise from septic shock. Sepsis and ALI share common mechanisms, including inflammation, endothelial dysfunction, and heightened permeability of alveolar epithelial cells [12]. Furthermore, septic shock is identified as the predominant cause of ALI, with patients experiencing sepsis-induced ALI exhibiting higher rates of mortality compared to individuals with alternative risk factors [13]. According to Paul (2018), the implementation of early targeted therapy in individuals diagnosed with septic shock has resulted in a decrease in the percentage of patients requiring mechanical ventilators [14].

Gallic acid is a phenolic acid that is found in many plants. The substance is a crystalline, colorless or slightly yellow substance often used in the food and drug industries [15]. According to previous research, gallic acid stops different bacteria from moving, sticking together, and making biofilms. These bacteria include *Pseudomonas aeruginosa*, *Staphylococcus aureus*, *Streptococcus mutans*, *Chromobacterium violaceum*, and *Listeria monocytogenes* [16]. Additionally, it has been shown that this substance can damage the structure of the cellular membrane, which can change the charge, hydrophobicity, and permeability of the membrane surface in both Gram-positive and Gram-negative bacteria [17]. Another thing it can do is damage the outer layer of Gram-negative bacteria [18]. The primary hypothesis of this study posits that the pretreatment of gallic acid is essential for activating these factors that contribute to the development of sepsis and, subsequently, acute lung injury (ALI).

Malondialdehyde (MDA) is an end product formed during the breakdown of cell membrane phospholipids during oxidative stress and lipid peroxidation [19]. Studies have shown that MDA levels, which are markers of lipid peroxidation, increased in the serum of patients with severe sepsis and were found to be related to sepsis severity [20].

Proinflammatory cytokines, including interleukin-1 (IL-1), interleukin-6 (IL-6), and tumor necrosis factor-alpha (TNF-alpha), play a critical role in the regulation of inflammation processes. During sepsis, when the body is fighting infection, overproduction of these cytokines can lead to an out-of-control immune response and the exacerbation of systemic inflammation. The involvement of TNF-a and IL-1 in sepsis has been extensively documented in various studies, encompassing both animal models of septic shock and investigations conducted on human subjects afflicted with sepsis [21,22].

During sepsis, there may be an unbalanced inflammatory response due to cytokine storm, and IL-1, IL-6, and TNF-alpha may be produced in excessive amounts and may increase the production of other proinflammatory cytokines [23]. During sepsis, IL-6 levels rise rapidly after TNF-α and IL-1 concentrations and play a vital role in the patient’s progression to multiorgan failure [23]. Extreme inflammatory conditions such as sepsis lead to lactate overproduction by accelerating glycolysis [24]. Increased lactic acid levels may reflect tissue hypoxia and oxygenation disorders and indicate circulatory disorders during sepsis [24]. C-reactive protein (CRP) is an acute phase reactant secreted by the liver during inflammation, and occupies an important place among these biomarkers [25].

The study conducted by Bora et al. [20] shows that mice exposed to fecal intraperitoneal injection procedure (FIP) exhibit a progressive systemic inflammatory syndrome accompanied by subsequent septic shock and multiorgan damage. The FIP model exhibits a cytokine profile closely resembling the cytokine profile observed in human cases of sepsis and the subsequent development of fulminant multiple organ failure [26].

This study aimed to investigate the effect of gallic acid pretreatment on the survival of septic animals, especially in areas where treatment needs to start quickly, such as the emergency department. This pretreatment has the potential to reduce inflammatory responses, organ damage, and mortality during the initial stages of sepsis induced by the FIP.

## 2. Materials and Methods

### 2.1. Animals

A group of 36 adult male Wistar albino rats, weighing an average of 200 to 250 g, were used in this study. The experiments in this study followed the guidelines in the Guide for the Care and Use of Laboratory Animals, which was made official by the National Institutes of Health in the United States. The Animal Ethics Committee at Science University approved the number 18210619. The rats used in the experiments came from Science University’s Experimental Animal Laboratory. The rats were free to consume food whenever they wanted. They lived in steel cages in controlled environments that kept the temperature at 22 ± 2 °C and changed the light and dark cycles every 12 h.

### 2.2. Experimental Procedures

We used the feces intraperitoneal-injection procedure (FIP) model for induced sepsis. The FIP rat model was established according to a method previously described by Shrum et al. and Tyml et al. [26,27]. For the FIP model, two adult rats were sacrificed by high-dose anesthesia and cervical dislocation. Fecal material was freshly prepared from the cecum and suspended in saline at 75 mg/mL. Then, the solution was injected with a 21G injector intraperitoneally (i.p.) at 1 g per kg body weight only one time. Rats were randomly assigned into four groups, and FIP was performed on 28 rats to induce the FIP sepsis model. Eight rats were divided into a normal group and no procedure. Four rats died during the first 24 h following the procedure and were excluded from the study. Study groups were designed as follows:

Group 1: Normal control group is non-operative and orally fed control (n = 8);
Group 2: FIP group,
Group 3: FIP and 10 mL/kg % 0.9 NaCl saline (placebo) group given by i.p. (n = 8).
Group 4: FIP and 20 mg/kg/day Gallic acid (Merk Sigma-Aldrich, Burlington, MA, USA) given by i.p. (n = 8).

All treatments were administered after one hour of the FIP model. At the end of the 20 h, all animals were given a CT procedure under ketamine anesthesia. The study was finished after 24 h. At the end of the study, euthanasia was performed with a high-dose anesthesia combination (100 mg/kg, Ketasol^®^ %10, Richter Pharma AG, Wels, Austria/xylazine 50 mg/kg, Rompun, Bayer, Germany) and cervical dislocation. Their blood samples were collected by cardiac puncture for biochemical analysis.

### 2.3. Determination of TNF-a, CRP, IL-6, IL 1-Beta, and Lactic Acid Levels in Plasma

TNF-a, CRP, IL 1-Beta, and IL-6 plasma levels were quantified using commercially available enzyme-linked immunosorbent assay (ELISA) kits from Biosciences and Abcam Cambridge, UK. The measurements were conducted in accordance with the guidelines provided by the manufacturer. The plasma samples were diluted at a ratio of 1:2, as per the guidelines provided by the manufacturer. Duplicate measurements were conducted to determine the levels of CRP and TNF-a. The determination of lactic acid levels was conducted utilizing a blood gas analyzer.

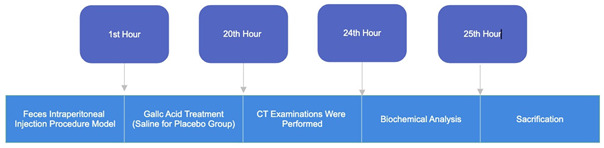



### 2.4. Measurement of Lipid Peroxidation

Lipid peroxidation was quantified in plasma samples by assessing malondialdehyde (MDA) levels as a thiobarbituric acid reactive substance. Concisely, trichloroacetic acid and TBARS reagent were introduced to the plasma samples, subsequently combined, and subjected to incubation at 100 °C for 60 min. After cooling on ice, the samples underwent centrifugation at a rate of 3000 revolutions per minute for 20 min. Subsequently, the absorbance of the resulting supernatant was measured at a wavelength of 535 nanometers [28].

### 2.5. Histopathological Examination of Lung

To perform histological analysis, all animals were anesthetized intraperitoneally with ketamine (100 mg/kg, Alfamine^®^, Alfasan International BV, Utrecht, Holland) and xylazine (50 mg/kg, Alfazyne^®^, Alfasan International BV Utrecht, Holland). They were then given 200 mL of a 4% formaldehyde solution intravenously in 0.1 M phosphate-buffered saline (PBS). The lung sections, which had been fixed in formalin and had a thickness of 5 m, were stained with hematoxylin and eosin (H&E). The images were taken with an Olympus C-5050 (Olympus Corp., Tokyo, Japan). A digital camera is attached to an Olympus (Olympus Corp., Tokyo, Japan). BX51 microscope. The primary histopathological lung damage score was calculated in accordance with previous research [20]. In summary, histopathological lung damage was assessed by quantifying various indicators such as alveolar congestion (AC), hemorrhage (H), leukocyte infiltration or aggregation in air spaces/vessel walls (AL), perivascular/interstitial edema (PE), and alveolar wall thickness/hyaline membrane formation (TA). The severity of each item was graded on a scale of 1 (0–25%), 2 (25–50%), 3 (50–75%), and 4 (75–100%) [20].

### 2.6. CT Examination of Lung

Following an anesthetic agent injection, the examinations were performed supine using a 16-slice multi-detector row CT scanner (Somatom Go Now, Siemens Healthcare, Erlangen, Germany) without administering contrast media. A ketamine–xylazine mixture was administered intraperitoneally to the rats to induce deep anesthesia. The ketamine dosage was 80 mg/kg from Richterpharma AG Austria, and the xylazine dosage was 10 mg/kg from Rompun by Bayer, Germany. All animals were securely immobilized on a scanning table using appropriate materials to minimize motion artifacts. The scanning parameters used in the study were 120 kilovolts (kV), variable milliampere seconds (mAs) determined by an automatic exposure control system, and 1 mm slice thickness. The scanning range extended from the C3 vertebrae to the diaphragm, including the apex and base of the lung. Following the image acquisition process, all images were reconstructed at a slice thickness of 1 mm to ensure non-overlapping slices. The reconstruction was performed with a 512 × 512 matrix and a sharp reconstruction kernel known as KernelBr64. Three radiologists (B.H.S. and S.G.G.) evaluated all images who needed to be made aware of the laboratory findings and animal group assignments. Six areas of interest (ROI) were chosen for investigation. Each ROI was precisely 2153 mm^2^. The ROIs were distributed evenly across the upper, middle, and lower zones of both lungs, with two in each zone. All animals’ axial images with a parenchymal window were taken near the heart apex. Precautions were taken to avoid placing the region of interest (ROI) near large blood vessels, airways, or bones.

### 2.7. Arterial Blood Gas Analysis

Blood samples (0.2 mL) were collected from the carotid artery of rats in each experimental group at 24 h post-operation. The collected blood samples were then analyzed using a blood gas analyzer to determine the levels of PaO_2_ and PaCO_2_.

### 2.8. Statistical Analysis

The analyses were conducted using the IBM SPSS Statistics Standard Concurrent User V 26 software package (IBM Corp., Armonk, New York, NY, USA). Numerical summaries were provided as mean ± standard error of the mean (SEM). For two or more categorical parameters, one-way analysis of variance (ANOVA) was employed if the dataset exhibited a normal distribution; otherwise, the Kruskal–Wallis H Test was utilized. If the Analysis of Variance results were statistically significant, the Bonferroni test was applied as a multiple comparison test. A significance level of *p* < 0.05 was accepted, and *p* < 0.001 was considered statistically highly significant.

## 3. Results

### 3.1. Biochemical Findings

Malondialdehyde (MDA) levels were 11.8 ± 1.3 in the normal control group, 40.9 ± 3.1 (*p* < 0.001) in the FIP group, 35.9 ± 4.4 (*p* < 0.001) in the FIP and saline group, and 16.5 ± 1.8 (*p* < 0.001) in the FIP and 20 mg/kg gallic acid group. Similarly, IL-6, IL-1-beta, TNF-alpha, and CRP levels were significantly increased in the FIP group, but these increases were significantly decreased in the FIP and 20 mg/kg gallic acid groups. Lactic acid levels were 4.05 ± 0.9 (*p* < 0.001) in the FIP group, 4.1 ± 0.8 (*p* < 0.05) in the FIP and saline group, and 2.03 ± 0.5 (*p* < 0.001) in the FIP and 20 mg/kg gallic acid group (Table 1).

**Figure 1:**
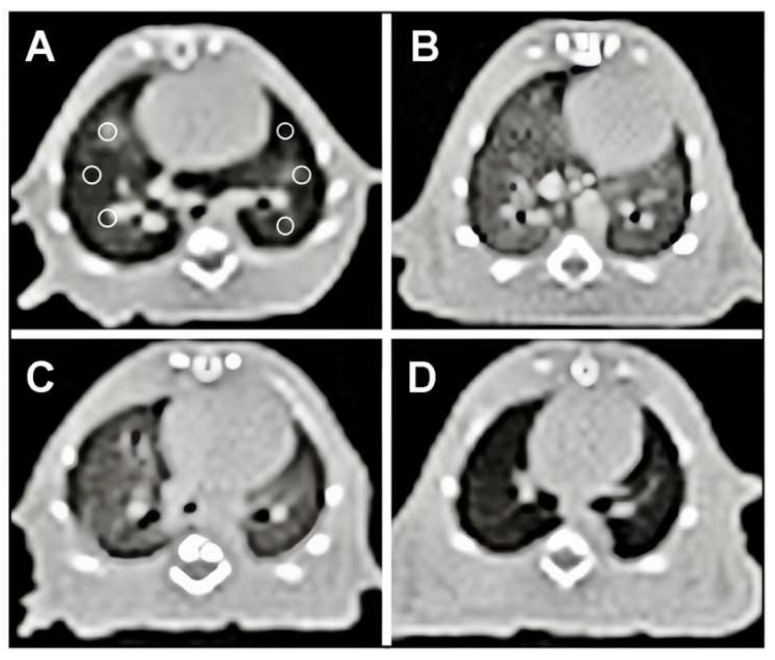
Axial CT images of the lung at the level of the heart, six ROI placed with the same size at the exact location (**A**) Normal Control group lung, (**B**) FIP group showed increased density of lung, (**C**) FIP and 10 mL/kg % 0.9 NaCl saline (placebo) group showed increased density of lung, (**D**) FIP and 20 mg/kg gallic acid group showed density of lung closer to the normal group.

**Figure 2:**
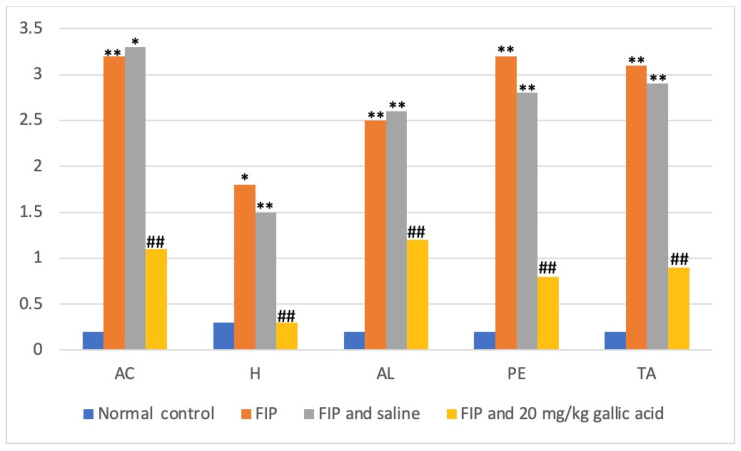
Results were presented as mean ± SEM. Statistical analyses were performed using one-way ANOVA. * *p* < 0.01, ** *p* < 0.0001 different from normal groups; ## *p* < 0.001 different from FIP and saline group. AC: alveolar congestion, H: hemorrhage, AL: Leukocyteinfiltrationoraggregationinair spaces/vessel walls, PE: Perivascular/interstitial edema.

The CT (computed tomography) Hounsfield unit (HU) values in the aortic aperture (AC), hypothalamus (H), amygdala (AL), prefrontal cortex (PE), and thalamus (TA) regions were 0.2 ± 0.1, 0.3 ± 0.2, 0.2 ± 0.1, 0.2 ± 0.1, 0.2 ± 0.1, and 0.2 ± 0.1, respectively. The values in the FIP group were 3.2 ± 0.2 (*p* < 0.0001), 1.8 ± 0.3 (*p* < 0.01 *), 2.5 ± 0.3 (*p* < 0.0001), 3.2 ± 0.1 (*p* < 0.0001), and 3.1 ± 0.3 (*p* < 0.0001), respectively. In the FIP and saline groups, these values were 3.3 ± 0.2 (*p* < 0.01), 1.5 ± 0.3 (*p* < 0.0001), 2.6 ± 0.2 (*p* < 0.0001), 2.8 ± 0.4 (*p* < 0.0001), and 2.9 ± 0.2 (*p* < 0.0001), respectively. In the FIP and 20 mg/kg gallic acid groups, the values in the AC, H, AL, PE, and TA regions were determined as 1.1 ± 0.2 (*p* < 0.001), 0.3 ± 0.1 (*p* < 0.001), 1.2 ± 0.1 (*p* < 0.001), 0.8 ± 0.3 (*p* < 0.001), and 0.9 ± 0.1 (*p* < 0.001), respectively (Figure 1 and Figure 2).

### 3.2. Arterial PaO_2_ and PaCO_2_

In the control group, arterial oxygen pressure (PaO_2_) was determined as 102.5 ± 7.3 mmHg. This value was 80.4 ± 9.1 mmHg in the FIP group, statistically significantly lower than the normal control group (*p* < 0.05). PaO_2_ in the FIP and saline group was 78.4 ± 3.4 mmHg, representing a low value compared to the normal control group (*p* < 0.05). In the FIP and 10 mg/kg gallic acid group, the PaO_2_ value was 97.1 ± 5.2 mmHg, which was significantly higher than the FIP and saline group (*p* < 0.05).

In terms of arterial carbon dioxide pressure (PaCO_2_), while the value was 40.8 ± 5.1 mmHg in the normal control group, this value was 31.7 ± 6.5 mmHg in the FIP group, which was significantly lower than the normal control group (*p* < 0.05). PaCO_2_ was 33.8 ± 2.9 mmHg in the FIP and saline group, which was lower than the normal control group but not statistically significant (*p* > 0.05). In the FIP and 10 mg/kg gallic acid group, PaCO_2_ was 35.3 ± 4.8 mmHg, and this value was significantly higher than the FIP and saline group (*p* < 0.05) (Table 2).

### 3.3. Histological Score and CT Findings

**Figure 3:**
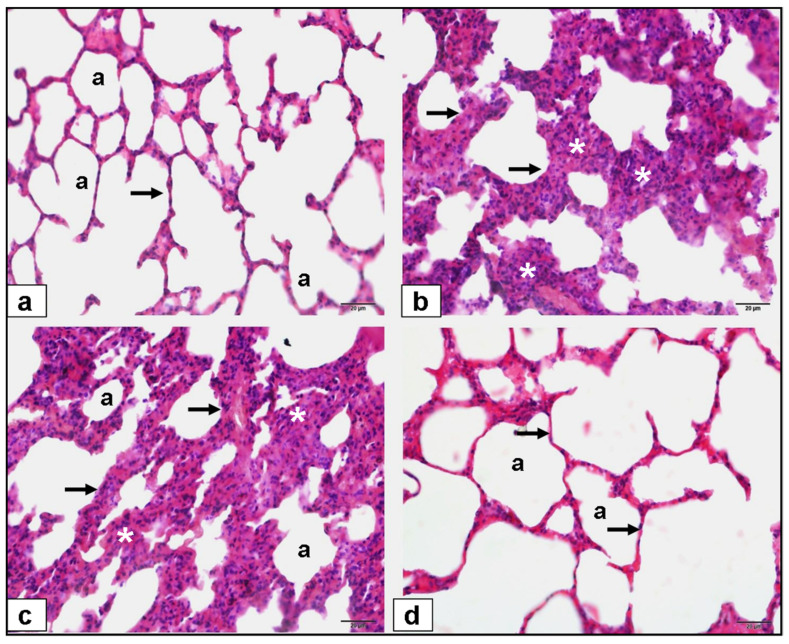
Lung histopathology 40× magnification H&E staining. (**a**) Normal control group lung, “a” Alvelol, (**b**) FIP groups showed severe histopathologic alteration related to increased alveolar inflammation (*) and septal thickness (arrow), (**c**) FIP and 10 mL/kg % 0.9 NaCl saline (placebo) groups showed severe histopathologic alteration related to increased alveolar inflammation (*) and septal thickness (arrow), (**d**) FIP and 20 mg/kg gallic acid groups showed decreased inflammation and septal thickening (arrow).

Lung Histopathology (H&E Staining) at 40× magnification: The histological images reveal the protective effect of gallic acid, evident in the attenuation of inflammatory changes and preservation of lung architecture compared to the FIP and saline (placebo) groups. The reduction in inflammation and septal thickness in the gallic acid-treated group suggests a potential therapeutic role in mitigating lung damage induced by FIP (Figure 3).

## 4. Discussion

Previous research has confirmed the importance of initiating antibiotic treatment as soon as possible in sepsis and septic shock cases, to prevent acute lung injury (ALI) and infection spread [20,29]. So far, there has been insufficient evidence to support the effectiveness of any pharmaceutical intervention in reducing the inflammatory response and maintaining hemodynamics during the initial phase. The findings of our current study provide empirical evidence to support the benefits associated with the early application of gallic acid in cases of ALI.

Gallic acid is naturally found in a variety of plant and fruit groups. Numerous studies on gallic acid have revealed that it possesses various potential therapeutic properties, including anti-cancer and antimicrobial properties. Furthermore, it is worth noting that gallic acid and its derivatives can act as antioxidants indirectly. This is accomplished by increasing the activity of antioxidant enzymes. The gallic acid administration resulted in a diminished MDA value, which had previously augmented following the experimental model. Similarly, in a study on liver damage in rats, Ojeaburu et al. discovered that MDA levels in rats treated with gallic acid were lower than in the control group [30].

In contrast, Maurya et al. [31] used gallic acid at a dose of 20 mg/kg orally in septic animals, and a reduction in MDA level was observed in the gallic acid + sepsis group. We administered gallic acid intraperitoneally at a dose of 20 mg/kg daily and found similar results. This finding suggests that the treatment effectively reduced tissue oxygen demand by reducing systemic oxidative stress.

CRP is reliable for detecting and assessing inflammation. Gallic acid’s CRP inhibition affects ALI and other inflammatory disease treatments [20]. Our study’s CRP reduction compared to the FIP and FIP + saline groups supported gallic acid’s anti-inflammatory properties. A lung inflammation study by Sarkaki et al. found that gallic acid significantly reduced CRP levels, supporting this finding. Sarkaki suggests that the liver regulates CRP release in addition to gallic acid’s anti-inflammatory effects [32]. Although CRP elevation alone does not indicate anything, it may be helpful with other findings.

Proinflammatory cytokines TNF-a, IL-1b, and IL-6 cause early inflammation. For sepsis severity assessment and patient monitoring, IL-6 measurement has become important [33,34]. In our study’s FIP procedure, TNF-a, CRP, IL-1b, IL-6, and MDA levels increased significantly in both FIP alone and FIP + saline groups. After gallic acid treatment, these levels dropped significantly. Kim et al. [33] found lower TNF-a and IL-6 levels in gallic acid-treated rats than controls. Sarkaki et al. [32] found that gallic acid administration reduced TNF-a, IL-1, and IL-6 in traumatic brain injury rats, which matches this study’s findings. Gallic acid may reduce systemic inflammation during sepsis, data suggests.

Serum lactate levels are thought to be an indicator of circulatory problems and rise during sepsis [35]. Lactate levels were found to be lower in the gallic acid-treated groups in this study. The regression of lactate levels with gallic acid suggests that it, in addition to antioxidant–antinflammatory mechanisms, regresses sepsis.

In the FIP group, histopathological examinations revealed severe lung damage and inflammation. These histopathological changes, however, were lower in the gallic acid-treated groups. This demonstrates the ability of gallic acid to protect lung tissue. In their study on rats, Hong et al. histopathologically demonstrated that gallic acid has a protective effect on kidney tissue, and that the same effect can be observed in different tissues [36]. Similar histopathological results with similar mechanisms were found in another study that evaluated the anti-inflammatory effect of digoxin [20], confirming the effectiveness of the antioxidant anti-inflammatory effect.

Bora et al. [20] obtained similar results in their assessment of the regions of interest (ROIs) in thoracic computed tomography (CT) using the Hounsfield unit, as shown in Figure 2—the use of computed tomography for imaging rats. Six equal-sized regions of interest (ROIs) were placed at the exact location on axial computed tomography (CT) images of the lung at the heart level. The lung of the normal control group is shown in panel A. The lung density of the FIP group is elevated in panel B. Similarly, the FIP + saline group has increased lung density in panel C. Panel D, moreover, shows that the lung density in the FIP + gallic acid group is similar to that of the normal group.

In a radiological study conducted by Gattinoni et al. [37], it was discovered that the ventilation in the digitoxin group was statistically higher than in the FIP and FIP + saline groups. This experiment’s findings suggest that digitoxin may benefit acute lung injury (ALI) by reducing the exaggerated inflammatory response associated with sepsis during the first phase. Tang et al. [38] discovered that the FIP group had low levels of arterial oxygen pressure (PaO_2_). Gallic acid-treated groups, on the other hand, had levels that were closer to normal. This finding supports the beneficial effect of gallic acid on lung tissue protection.

## 5. Conclusions

The therapeutic properties of Gallic acid have been documented through a range of evaluations, encompassing histological and radiological examinations of the lungs and biochemical analysis of its healing effects in cases of ALI and sepsis. Incorporating gallic acid, in conjunction with the prescribed treatments outlined in the guidelines, at the initial manifestation of sepsis may serve as a protective measure against adverse effects, such as acute lung injury (ALI). Furthermore, this approach could decrease mortality rates and mitigate the necessity for mechanical ventilation by averting the occurrence of a cytokine storm.

## 6. Limitations

The limitations of this study are that it is an animal experiment, and the ethical effects on humans may differ. Another limitation is that the doses of gallic acid applied should be evaluated across a broader spectrum. On the other hand, different doses of gallic acid should be tried for better results.

## Figures and Tables

**Table 1:** Results were presented as mean ± SEM. Statistical analyses were performed using one-way ANOVA. * *p* < 0.05, ** *p* < 0.001 different from normal groups; ## *p* < 0.001 different from FIP and saline group.

	Normal Control	FIP	FIP and Saline	FIP and 20 mg/kg Gallic Acid
MDA (nM/mg protein)	11.8 ± 1.3	40.9 ± 3.1 **	35.9 ± 4.4**	16.5 ± 1.8 ##
IL-6 (pg/mL)	5.8 ± 0.9	23,849.2 ± 1496.2 **	25,661.5 ± 1378.6 **	10,325.7 ± 985.2 ##
IL 1-Beta (pg/mL)	2.5 ± 0.1	2025.3 ± 66.5 **	1945.8 ± 103.4 **	365.2 ± 29.07 ##
TNF alfa (pg/mL)	14.2 ± 1.9	401.9 ± 12.8 **	421.5 ± 18.9 **	185.1 ± 28.3 ##
CRP (mg/dL)	0.45 ± 0.3	1.19 ± 0.3 *	1.22 ± 0.2 *	0.7 ± 0.2 ##
Lactic acid (mmol/L)	1.1± 0.2	4.05 ± 0.9 **	4.1 ± 0.8 *	2.03 ± 0.5 ##

**Table 2:** Results were presented as mean ± SEM. Statistical analyses were performed using one-way ANOVA and a post hoc Bonferroni test. * *p* < 0.05, different from normal groups; # *p* < 0.05 different from FIP and saline group.

	Normal Control	FIP	FIP and Saline	FIP and 10 mg/kg Gallic Acid
PaO_2_ (mmHg)	102.5 ± 7.3	80.4 ± 9.1 *	78.4 ± 3.4 *	97.1 ± 5.2 #
PaCO_2_ (mmHg)	40.8 ± 5.1	31.7 ± 6.5 *	33.8 ± 2.9 *	35.3 ± 4.8

## Data Availability

Data available if there is an appropriate reason.
