# Correction: Kardaş et al. Gallic Acid Protects from Sepsis-Induced Acute Lung Injury. *Curr. Issues Mol. Biol.* 2024, *46*, 1–10

**DOI:** 10.3390/cimb47080669

**Published:** 2025-08-19

**Authors:** Süleyman Kardaş, Osman Sezer Çınaroğlu, Ejder Saylav Bora, Oytun Erbaş

**Affiliations:** 1 Department of Emergency Medicine, Kızıltepe State Hospital, Mardin 47400, Türkiye; 2 Department of Emergency Medicine, Faculty of Medicine, Izmir Katip Çelebi University, Izmir 35270, Türkiye; drsezer@hotmail.com (O.S.Ç.); saylavbora@hotmail.com (E.S.B.); 3 Department of Physiology, Faculty of Medicine Demiroğlu Science University, Istanbul 34000, Türkiye; oytunerbas2012@gmail.com

## Error in Figure

In the original publication [1], the authors unfortunately made a mistake in Figure 1 as published when configuring and uploading the figures. None of the authors noticed that the figures were unintentionally mixed up in the final proofreading before publication. The authors identified the regrettable mistake and requested that it be rectified, replacing the inappropriate Figure with the correct one. The corrected Figure 1 appears below. The authors state that the scientific conclusions are unaffected. This correction was approved by the Academic Editor. The original publication has also been updated.

## Figures and Tables

**Figure 1:**
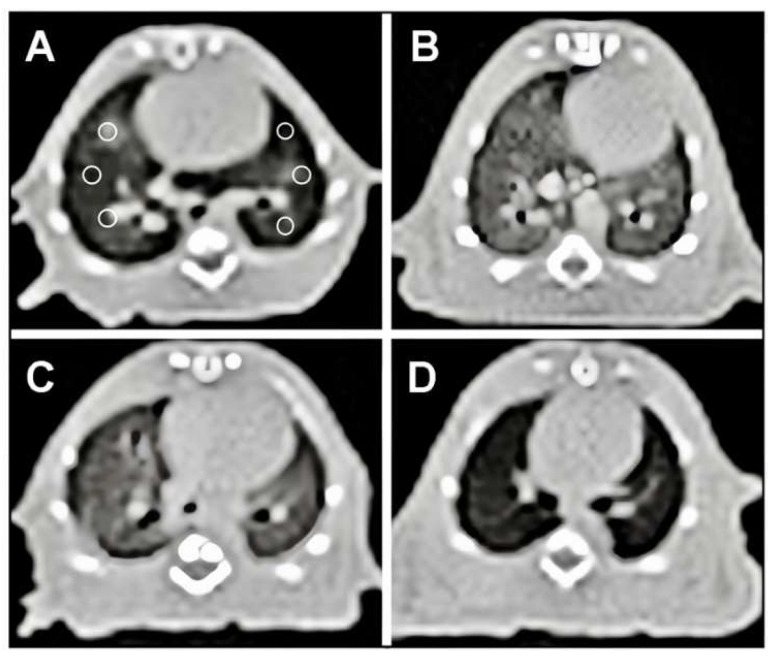
Axial CT images of the lung at the level of the heart, six ROI placed with the same size at the exact location (**A**) Normal Control group lung, (**B**) FIP group showed increased density of lung, (**C**) FIP and 10 mL/kg % 0.9 NaCl saline (placebo) group showed increased density of lung, (**D**) FIP and 20 mg/kg gallic acid group showed density of lung closer to the normal group.
